# Testing the generalization of neural representations

**DOI:** 10.1016/j.neuroimage.2023.120258

**Published:** 2023-09

**Authors:** Florian Sandhaeger, Markus Siegel

**Affiliations:** a Department of Neural Dynamics and Magnetoencephalography, Hertie Institute for Clinical Brain Research, University of Tübingen, Germany; b Centre for Integrative Neuroscience, University of Tübingen, Germany; c MEG Center, University of Tübingen, Germany; d IMPRS for Cognitive and Systems Neuroscience, University of Tübingen, Germany

**Keywords:** Representational generalization, Cross-decoding, Neural population measurements, Neural representations, Pattern generalization

## Abstract

Multivariate analysis methods are widely used in neuroscience to investigate the presence and structure of neural representations. Representational similarities across time or contexts are often investigated using pattern generalization, e.g. by training and testing multivariate decoders in different contexts, or by comparable pattern-based encoding methods. It is however unclear what conclusions can be validly drawn on the underlying neural representations when significant pattern generalization is found in mass signals such as LFP, EEG, MEG, or fMRI. Using simulations, we show how signal mixing and dependencies between measurements can drive significant pattern generalization even though the true underlying representations are orthogonal. We suggest that, using an accurate estimate of the expected pattern generalization given identical representations, it is nonetheless possible to test meaningful hypotheses about the generalization of neural representations. We offer such an estimate of the expected magnitude of pattern generalization and demonstrate how this measure can be used to assess the similarity and differences of neural representations across time and contexts.

## Highlights

• Multivariate pattern generalization is commonly used to assess the similarity of neural representations between contexts.
• When applied to neural mass signals such as LFP, MEG or fMRI, pattern generalization is susceptible to confounds due to spatial mixing.
• Statistically significant pattern generalization alone is not sufficient to conclude representational overlap.
• Using appropriate precautions, pattern generalization nonetheless enables valuable insights.

## 1 Introduction

**Fig. 1:**
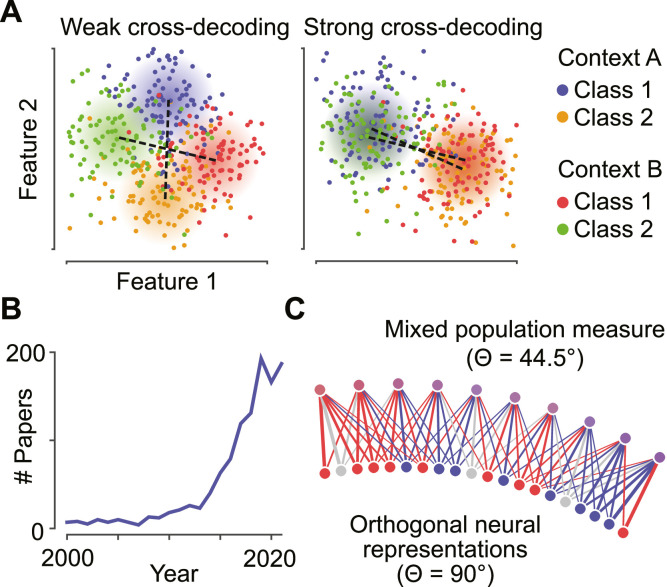
*Assessing the generalization of neural representations.***(A)** Illustration of pattern generalization. The difference vector of multivariate neuronal measurements between two classes (1 and 2) is determined in two contexts (A and B). If the difference vectors are aligned in both contexts, there is strong pattern generalization; if they are orthogonal there is no pattern generalization. The left scatterplot shows an example of weak negative pattern generalization, the right scatterplot an example of strong negative pattern generalization. **(B)** The use of pattern generalization techniques in neuroscience is rapidly increasing. **(C)** Spatial smoothing introduces similarity. Bottom, two distinct sets of neurons supporting two orthogonal representations (red and blue, respectively). Top, measurement of the same population with spatial mixing (e.g. by population measurement sensors). The measured representations are no longer orthogonal, which would result in significant pattern generalization if computed from the population signal.

Is the neural representation of a behavioral variable stable across time? Does it change between contexts or experimentally manipulated conditions? Are two variables represented in neural activity in similar ways? Multivariate pattern generalization methods offer a straightforward way to test such questions. For example, building on widely used decoding methods, a simple logic can be applied: if a decoding algorithm trained on neural data from one context works well when tested on data from another context, the representations of the variable in question in both contexts are related (Fig. 1A, Kaplan et al., 2015; King and Dehaene, 2014; Kriegeskorte and Douglas, 2019. For definitions of key concepts, see Table 1). Consequently, an identical neural readout mechanism could extract meaningful information in either case. This interpretation is evident when the measurement level matches the relevant biological scale: when cross-decoding is successfully applied to the spiking activity of individual neurons, it is plausible that a similar readout is implemented in the brain, and that the identified overlap of neural representations has an effect on neural computation and behavior.

**Table 1:** Glossary of core definitions.

Concept	Definition
Class	A set of trials defined by the value of a variable we want to quantify neural information about. This can e.g. be done using a classifier, by assessing the accuracy of predicted class labels.
Context	Any change in the experimental circumstances. In pattern generalization analysis, we want to quantify whether the representations underlying neural information are invariant across contexts. Examples of context variables include time, or any experimentally manipulated condition.
Decoding	In decoding analyses, class labels are predicted from the neural data. A common example are classifiers which predict the class label of each trial.
Encoding	In encoding analyses, aspects of neural data are predicted from the class labels. For example, the cross-validated Mahalanobis distance quantifies the pattern separation between trials of two classes.
Identity	Here, we consider two neural representations identical if the vectors separating the two classes of each representation are perfectly collinear.
Neural information	Any measure indicating the degree of reliable separation of neural activity patterns between classes. For our purposes, neural information can be quantified both by decoding (e.g. classifier accuracy) or encoding measures (e.g. Mahalanobis distances).
Neural representation	Here, the neural representation of a variable is the difference in the neural activity pattern between values of that variable. Thus, we define neural representations in their most general sense.
Pattern generalization	The degree to which the neural activity pattern differences between two classes are shared across two contexts. In other words, pattern generalization describes the similarity of two neural representations. Pattern generalization can be assessed using either encoding (e.g. cross-classification between trials of two contexts) or decoding measures (e.g. based on cross-validated Mahalanobis distances with training and test data from different contexts).
Population level	The spatial scale at which a neural population measurement encompassing the activity of many neurons is taken, using for example MEG or fMRI.
Representational level	The spatial scale at which neural representations are implemented. Depending on the question at hand, this could for example be at the scale of neurons, cortical columns, or areas.
Stability	The tendency of neural representations to be shared across repetitions of the experiment. The neural representation of stimulus hemifield, for example, would be strongly stable across participants, recruiting the contralateral visual cortex.
Uniformity	The tendency of neural activity pattern differences between classes to have the same sign across neurons. For example, high contrast stimuli may be expected to elicit higher activity than low contrast stimuli in most neurons.

However, the pattern generalization framework is frequently, and increasingly, applied to neural data on different measurement scales (Fig. 1B), from single cell electrophysiology (Bernardi et al., 2020; Cavanagh et al., 2018; Maggi and Humphries, 2022; Minxha et al., 2020; Qasim et al., 2019; Sarma et al., 2016; Spaak et al., 2017; Stokes et al., 2013), via local field potentials (LFP), electrocorticography (ECoG, Kragel et al., 2017; Norman et al., 2019), electroencephalography and magnetoencephalography (EEG or MEG, Brandman et al., 2019; Carlson et al., 2013; King et al., 2016; Kok et al., 2017; Quentin et al., 2019; Sandhaeger et al., 2019; Strauss et al., 2015; Teichmann et al., 2019, 2018; Wolff et al., 2017), to functional magnetic resonance imaging (fMRI, Gallivan et al., 2011; Hindy et al., 2016; Jung et al., 2018; Thavabalasingam et al., 2019; Tsantani et al., 2019; van Loon et al., 2018; Vetter et al., 2014; Walther et al., 2011; Wang et al., 2013; Woo et al., 2014). In many cases there is a mismatch between the scale of the representations in question and the measurements taken to compare them: EEG electrodes, voxels or magnetometers have no biological relevance and merely serve to sample aggregate measures of neural population activity. Despite this mismatch, significant pattern generalization in population measures is often interpreted strongly to indicate the generalization of the underlying neural representations (Aller and Noppeney, 2019; King and Dehaene, 2014; Levine and Schwarzbach, 2018; Sanchez et al., 2020; Teichmann et al., 2019, 2018; Zubarev and Parkkonen, 2018).

Pattern generalization is also used to test other hypotheses. In principle, pattern generalization can provide a graded measure of representational overlap, and not just the basis for a binary decision about its presence. Comparing pattern generalization between contexts with the strength of the individual patterns within each context enables testing whether representations are significantly different, which has for example been used to establish temporal dynamics (Myers et al., 2015; Spaak et al., 2017). Furthermore, it can not only be tested whether representations are overlapping at all, but also whether they are overlapping more than expected in a neural population with random selectivity (Bernardi et al., 2020). Pattern generalization has also been proposed to provide a general framework for the evaluation of abstraction in neural circuits (Bernardi et al., 2020).

Despite this broad application, it is unclear under which conditions, and to what extent, such interpretations of pattern generalization in neural mass data are valid. Here, we address these questions. We first simulate measurements of neural population activity to show how consistencies of the underlying representations lead to spurious pattern generalization in mass signals. We then introduce a measure of expected pattern generalization under the assumption of identity, which can be used to test against the null hypothesis of identical representations and serves as a benchmark for empirical pattern generalization values. We illustrate the interpretational caveats of pattern generalization for current neuroscientific practice using simulated and real MEG data. Finally, we provide practical recommendations for the interpretation of pattern generalization results.

The interpretational pitfalls we identify in the present work affect measures of pattern generalization based on both decoding methods (such as cross-classification algorithms) and encoding methods (such as the cross-validated Mahalanobis distance, or cross-validated MANOVA, Allefeld and Haynes, 2014; Christophel et al., 2018; Diedrichsen et al., 2016; Walther et al., 2016). For simplicity, in the following we do not differentiate between decoding- and encoding-based measures of representational strength and generalization unless necessary, and subsume them under the general terms of *neural information* and *pattern generalization*, respectively.

## 2 Results

Whether significant pattern generalization reliably indicates overlapping neural representations depends on the, often only partially known, relationship between these representations and the experimental measurement (Cichy et al., 2015; Cohen, 2017; Sandhaeger et al., 2019), including the sampling of neurons and signal mixing: When two representations are orthogonal in a neural population, the spatial smoothing inherent in neural mass recordings introduces spurious similarities between them (Fig. 1C). Consider a case where a single measurement sensor is used, reflecting average activity over the whole brain. Any two variables that have an effect on the measured data will either lead to an increase or decrease in activity, such that a pattern generalization analysis would always find them either positively (if both effects go in the same direction) or negatively (if they go in opposite directions) related. This would be the case regardless of whether the underlying neural representations recruit overlapping or fully orthogonal populations, or even distinct brain areas. With more measurement sensors, and less severe mixing, this effect would be weaker, but nonetheless present. Importantly, all modalities of neural mass data, including EEG, MEG, fMRI and even local field potentials, are affected by signal mixing and thus susceptible to this effect.

Does the reduction of orthogonality due to signal mixing render pattern generalization analyses on the population level invalid? Not necessarily: pattern generalization is typically not assessed on a single measurement or subject, but significance is determined statistically over a number of different subjects (or any other type of biological replicate). As long as the effects that mixing asserts on subjects are independent, there is no issue. The spurious correlation between mixed patterns would be positive in some subjects, and negative in others, resulting in pattern generalization and reversals respectively. Across the population this would average out, such that no consistent effect would be detectable.

However, it cannot generally be assumed that mixing affects each subject independently. For example, in fMRI there is an ongoing debate about the dominant sources of information in the BOLD signal (Carlson, 2014; Formisano and Kriegeskorte, 2012; Freeman et al., 2013, 2011; Roth et al., 2022, 2018). While the functional selectivity of voxels may be partially determined by a random sampling of the underlying neuronal population, in many circumstances maps or biases on a larger spatial scale may contribute to fMRI decoding. Similar, and arguably stronger, considerations apply in EEG and MEG (Cichy et al., 2015). If such maps exhibit a substantial similarity between individuals, and thereby violate the independence of multivariate patterns, pattern generalization caused by spatial mixing may become consistent over seemingly independent subjects. Consequently, this yields the impression that representations are overlapping when they are in fact not.

### 2.1 Spurious pattern generalization due to consistent mixing effects over replicates

**Fig. 2:**
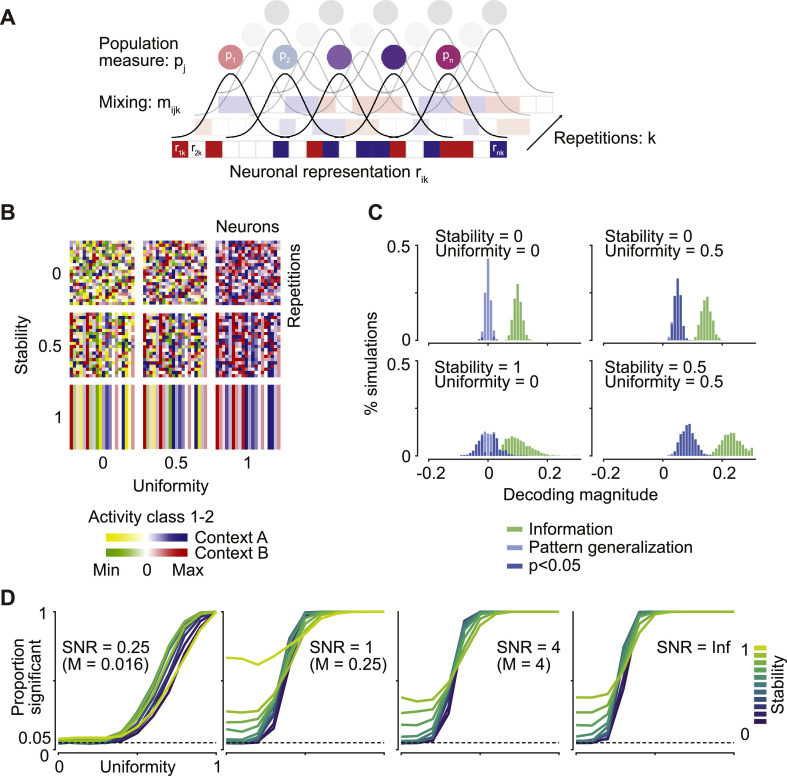
*Spurious pattern generalization due to consistent mixing.***(A)** Illustration of the simulation. Orthogonal representations were constructed, and spatial mixing was applied to yield a population measure. **(B)** We evaluated spurious pattern generalization as a function of uniformity (i.e. the tendency of class-differences to have the same sign) and stability (i.e. the tendency of representations to be similar across subjects). **(C)** Distribution of neural information and pattern generalization for four example parameter combinations, at an SNR of 1. **(D)** Proportion of simulations with significant pattern generalization (*p* < 0.05) for different SNRs, uniformity and stability. The three finite SNR conditions correspond to Mahalanobis distances (M) of 0.0156, 0.25 and 4.

To investigate how signal mixing in combination with a consistent relation between measurement sensors and the underlying representations leads to spurious pattern generalization, we implemented a simulation that allowed us to quantify the effect of several parameters (Fig. 2A). For each of two contexts, we first assigned random weights discriminating between two classes of trials to distinct subsets in a total of 100 neurons. We used these weights to simulate responses in 1000 trials per context by multiplying them with a signal-to-noise ratio (SNR) and adding Gaussian noise. We spatially mixed the responses of all neurons into 10 population measures using Gaussian mixing functions with a standard deviation of 25 neurons. In each simulation run, we generated these population responses 20 times, in order to create a dataset similar to those commonly used in neuroimaging. In a standard neuroscientific experiment, these repetitions would correspond to participants, while the population measure itself may constitute LFP, fMRI, EEG or MEG data. Crucially, the two subsets of neurons corresponding to each context were always non-overlapping, that is, no neuron showed activity differentiating between the classes in both contexts. Thus, the class representations in both contexts were orthogonal, such that any pattern generalization would be spurious. We then applied a pattern generalization analysis by computing the cross-validated Mahalanobis distance between classes, using trials from one context as training-, and from the other context as test data in a two-fold cross-validation scheme. As a consequence of how the data were generated, each SNR condition corresponded to a specific average Mahalanobis distance between classes. In each simulation run we determined whether consistent pattern generalization was present across repetitions using standard t-statistics and a significance threshold of *p* < 0.05. Finally, we repeated the simulation 1000 times to estimate the false positive rate.

The properties of each multivariate pattern were defined by two variables (Fig. 2B): first, its uniformity, i.e. the tendency of class differences to have the same sign in each neuron. For a uniformity of 1, selective neurons always showed stronger activation for class A than for class B, while for a uniformity of 0, activation differences were symmetrically distributed around 0. Secondly, we defined the stability of the multivariate patterns over repetitions (e.g., subjects). When stability was 1, all repetitions shared the same multivariate patterns, whereas with a stability of 0, patterns were fully independent between repetitions.

When activation differences between the two classes in both contexts were symmetrically distributed around 0 (uniformity = 0) and fully independent between repetitions (stability = 0), there was strong neural information but little spurious pattern generalization (Fig. 2c, top left). We next parametrically modified the uniformity and stability of the simulated representations, and repeated the simulation with different SNR values. When representations were independent across repetitions (stability = 0) and non-uniform (uniformity = 0), at an alpha level of 5%, 5% of simulations resulted in significant pattern generalization which exactly matches the expected false positive rate (Fig. 2C and D). However, increases in either uniformity or in stability led to an inflation of the false positive rate. This inflation was quicker for higher SNRs. In extreme cases of high uniformity or stability, the false positive rate approached 100%, indicating that spurious pattern generalization would be found in every single case.

These results highlight an underlying assumption of the common practice of performing statistical tests of pattern generalization on the group level: for the outcome of these tests to be valid, all participants’ pattern generalization values must be measured independently of each other. This assumption is no longer fulfilled when the two representations to be compared are to some extent stable over repetitions, which may lead to spurious pattern generalization that is consistent across repetitions (e.g., subjects). Thus, the mere presence of significant pattern generalization in neural mass signals is not a sufficient indicator of overlapping representations at the underlying neural level.

### 2.2 Testing the identity of representations

While the effect of mixing complicates inferences about the presence of a representational overlap from pattern generalization in mass signals, pattern generalization analyses have also been used to test the complementary null-hypothesis of identical representations. In this context, two representations are considered identical when their defining multivariate pattern difference vectors are perfectly collinear. How does signal mixing affect such tests? If the pattern generalization between two contexts is lower than a certain threshold, it is concluded that the representations in both contexts are not identical. Importantly, this inference is not impacted by the mixing inherent in neural mass signals, which can only increase the similarity of representations. The validity of tests against the identity of representations thus extends to the underlying neural signals: if representations are found to be different, there has to be an underlying difference on the neural level. Such tests against the identity of representations have prominently been used to assess the limits of temporal generalization. By training a decoder at one time point, testing it at another, and finding that it does not generalize perfectly, some degree of temporal dynamics of the underlying representation can be established (Myers et al., 2015; Spaak et al., 2017).

**Fig. 3:**
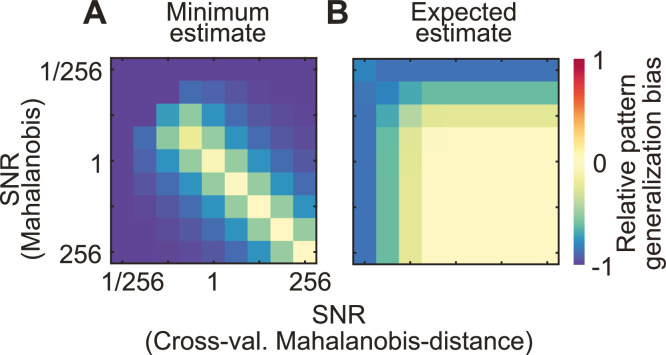
*Bias in the estimation of expected pattern generalization between contexts.***(A)** Using the minimum information magnitude as an estimate leads to a significant under-estimation when SNR is different in both contexts. **(B)** The novel expected pattern generalization estimate under identity introduced here provides a tighter lower bound, with little underestimation for medium to high SNRs. It works well when SNRs are different in both contexts. Bias is calculated as (estimated-true)/true pattern generalization.

An appropriate reference value to test the identity of representations using the pattern generalization between two contexts should consider the neural information within each context: if the two representations to be compared are both strong, we would expect stronger pattern generalization than if one or both are only weakly detectible. For cross-decoding analyses, a commonly used reference value is the minimum of the decoding values in both contexts (Myers et al., 2015; Spaak et al., 2017). More generally, any encoding- or decoding-measure of pattern distinctness can be used to compute a minimum information value. This works well when SNR – and consequentially the neural information – is similar in both contexts. However, in situations of unequal SNR, the minimum information value strongly under-estimates the true pattern generalization between identical representations. Thus, in such situations, tests against the identity of representations would often fail, leading to an elevated false negative rate (Fig. 3A).

It would therefore be desirable to test against a different reference value. Using the neural information about both representations, we can estimate a lower bound of the pattern generalization expected between both representations if they were identical. Importantly, while identity indicates that the vectors separating the two classes of each representation are perfectly collinear, their length may vary between representations. Thus, there may be different amounts of neural information about both representations. The computation of this expected pattern generalization under the assumption of identity is based on the geometric mean of both information values (see Appendix A for a complete derivation of the expected pattern generalization), and provides an accurate estimate for unbiased, symmetric information measures based on vector multiplication between training- and test data. This includes distance measures such as the cross-validated Mahalanobis distance, or the cross-validated MANOVA. Importantly, it is not valid for classifier accuracy, which involves a nonlinearity impairing the interpretability of cross-classification accuracies.

To validate this measure of expected pattern generalization under identity, we again simulated data from two classes in two contexts, this time enforcing the identity of representations between contexts. We then used the neural information values of both contexts to predict pattern generalization between them. We defined the bias of the predicted pattern generalization as the normalized difference between estimated and true pattern generalization. As theoretically expected, the estimate of expected pattern generalization provided a lower bound for true pattern generalization (Fig. 3B). For medium to high signal-to-noise ratios (here defined as the average Mahalanobis distance between classes), expected pattern generalization values were close to ground truth, and, in very low SNR simulations, they under-estimated the true pattern generalization. The underlying cause of this bias is the variability of the estimated neural information; for a given number of trials it scales monotonously with SNR. The extent of the bias in empirical data therefore corresponds to simulations with comparable sample sizes and Mahalanobis distances. Crucially, the measure proposed here offers a tighter lower bound than the previously suggested minimum of both neural information values (Fig. 3A vs. 3B). It can therefore be used for more sensitive tests against the identity of representations.

Finding that empirical pattern generalization values are significantly smaller than the expected pattern generalization under identity thus leads to the valid inference that representations are not identical. Notably though, the reverse test is not possible: within the framework of null-hypothesis significance testing, we can never conclude that the similarity between two representations is sufficiently close to the expected pattern generalization value to render them identical. Furthermore, being a lower bound, expected pattern generalization also does not offer the possibility to quantify evidence for the null hypothesis in a Bayesian framework.

### 2.3 Interpreting relative pattern generalization

So far, we have established that, first, the interpretation of the presence of pattern generalization from neural mass signals is complicated by mixing effects. Second, we have shown that the magnitude of pattern generalization one would expect if two representations were identical can be estimated, and that the deviation from identity can be statistically tested without interpretational problems. This measure of expected pattern generalization provides a crucial additional benefit by enabling the assessment of relative pattern generalization magnitudes. Raw pattern generalization magnitudes are difficult to interpret: the same pattern generalization values may, for example, be due to either a moderate representational overlap in a high SNR situation, or due to identical representations in a low SNR situation. Putting pattern generalization values in relation to the reference value of identical representations helps resolve such ambiguities. Importantly, the relative magnitude of pattern generalization values also constrains the possible sources of pattern similarity. While weak pattern generalization may often be spurious, strong pattern generalization approaching the expectation under identity indicates that the underlying neural representations are sufficiently similar to be indistinguishable by the measurement method.

In fMRI, for example, weak spurious pattern generalization could be found between two representations in distinct parts of an area, whereas near-identical pattern generalization is only plausible if the neural representations match at the voxel- or sub-voxel-level. Similarly, in MEG, weak spurious pattern generalization may occur between representations in distinct brain areas, as long as there is any measurement cross-talk between them, whereas strong pattern generalization would indicate matching patterns at the method's maximal spatial resolution on the order of millimeters or better (Cichy et al., 2015). In general, spurious pattern generalization values would, while significant, be far from those expected between identical representations. Therefore, the relative strength of pattern generalization can aid interpretation: weak, but significant pattern generalization is more likely to be spurious than strong pattern generalization. Importantly, this can only serve as a qualitative strategy. While the relative strength of pattern generalization may directly map onto a researcher's confidence in the result, there is currently, to our knowledge, no method to quantify this.

### 2.4 Spurious pattern generalization between orthogonal representations in simulated MEG data

To investigate if spurious pattern generalization could plausibly have a detrimental effect in typical brain imaging studies and to assess the usefulness of expected pattern generalization, we simulated neural responses in two classes of trials, in two different contexts. For each context, we defined a small set of neural sources to show increased activity in one of the two classes. Importantly, the class representations in both contexts were orthogonal. In this simple example, we constrained the representations to be uniform: every selective source in either context always showed stronger activity for class A than for class B. We added independent Gaussian noise to each trial's and source's activity and then used empirical forward models based on structural MRI scans of 19 human participants to project the simulated data to 272 MEG sensors. While noise was independent across participants, we used identical neural representations for each participant. This simulation thus corresponds to a situation where class differences are strongly dependent on an underlying topography that is stable across participants and driven by uniform activity differences between classes. A plausible example may be the presentation of weak (class 1) and strong (class 2) visual (context 1) and auditory (context 2) stimuli.

**Fig. 4:**
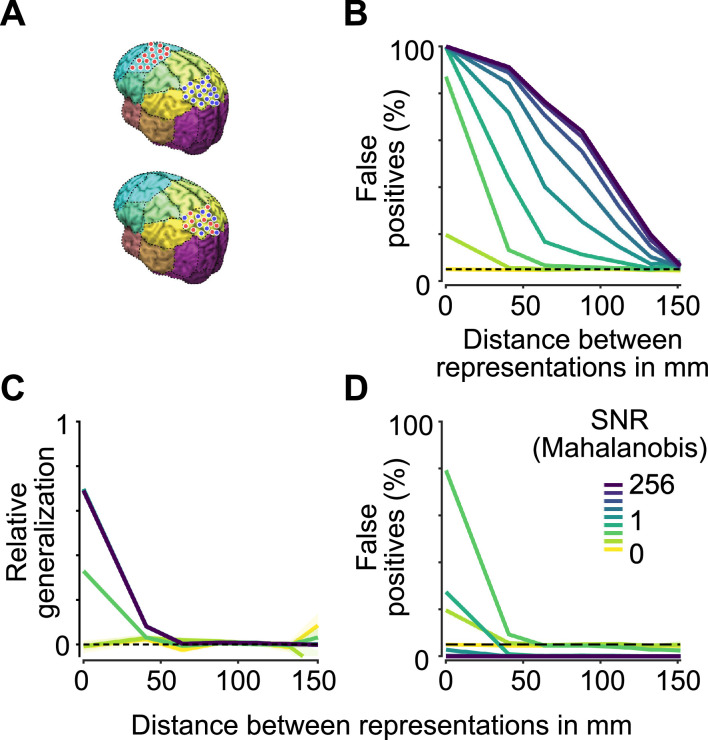
*Spurious pattern generalization in simulated MEG data.***(A)** Non-overlapping representations were placed in two out of 30 distinct brain areas. Top, example of two representations in distant areas (corresponding to a distance > 0 mm in B, C and D). Bottom, example of two representations in the same area (corresponding to a distance of 0 mm in B, C and D), which are nonetheless non-overlapping and therefore orthogonal. **(B)** Percentage of statistically significant pattern generalization results between orthogonal representations for different signal-to-noise ratios (SNR, corresponding to the cross-validated Mahalanobis distance between classes) and cortical distances. Representations were simulated on the source level, projected to MEG sensors, and pattern generalization analysis was applied. As representations are non-overlapping, significant results indicate false positives. **(C)** Spurious pattern generalization magnitudes in simulated MEG data relative to the expected pattern generalization between identical simulations. When representations are not placed in the same area, pattern generalization is very small. **(D)** Percentage of statistically significant pattern generalization results between orthogonal representations, which at the same time are *not* significantly smaller than expected, were they identical. Apart from low SNRs in simulations with representations in the same area, this effectively controls the false positive rate. High pattern generalization values can thus reliably indicate overlapping, or at least spatially very close, representations.

Again, we applied a pattern generalization analysis to the simulated population data using cross-validated Mahalanobis distances and a two-fold cross-validation scheme: for each simulation, we multiplied the vectors discriminating trial-classes in both contexts, which is comparable to training a decoder in one context and testing it in the other. To assess how the results depended on spatial distance between the representations in both contexts, we placed each representation in one of 30 cortical areas covering the whole brain and repeated this analysis for every pair of areas (Fig. 4A). Importantly, even when both representations were placed in the same area, their respective sets of selective sources were non-overlapping. Thus, any pattern generalization found would be spurious.

**Fig. 5:**
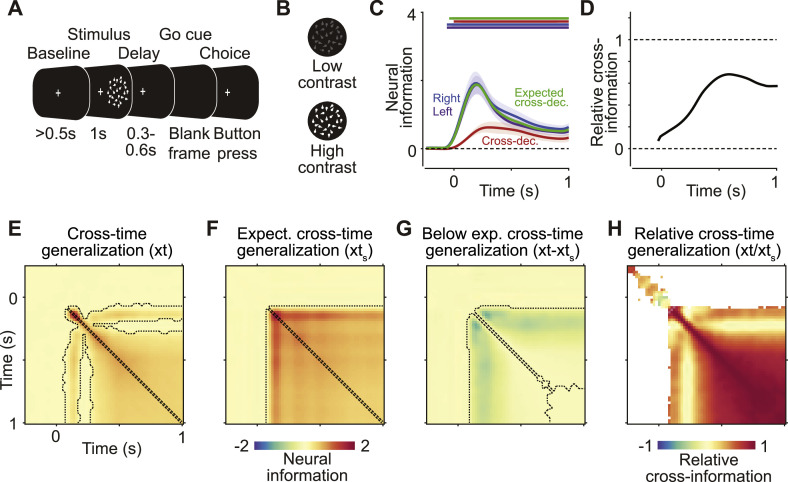
*Pattern generalization of contrast in example MEG data.***(A)** Behavioral task. Participants viewed dynamics random dot patterns with varying levels of contrast, in either the left or right hemifield, and reported the direction of motion. **(B)** We analyzed data from two contrast levels. **(C)** Pattern generalization (red) of the contrast of stimuli presented in the left (purple) and right (blue; note that the blue line is mostly obstructed by the nearly identical purple line) hemifields, as well as the expected pattern generalization if left- and right-hemifield representations were identical (green). Horizontal bars indicate significant clusters of information (blue and purple, *P* < 0.05, cluster permutation, one-tailed, *N* = 19), pattern generalization (red, two-tailed), or significantly smaller pattern generalization than expected (green, one-tailed). Coloured lines and shaded regions indicate the mean +/- SEM across participants. **(D)** Relative pattern generalization of contrast, calculated as empirical pattern generalization divided by expected pattern generalization under identity. **(E)** Mean cross-temporal generalization of contrast information. Dashed outlines indicate significant clusters of information (*P* < 0.05, one-tailed, cluster permutation, *N* = 19. **(F)** Mean expected cross-temporal generalization under the assumption of a perfectly stable representation. Dashed outlines indicate significant clusters of expected generalization > 0 (*P* < 0.05, cluster permutation, two-tailed, *N* = 19. **(G)** Difference between real (panel F) and expected (panel G) cross-temporal generalization, indicating a dynamic representation of contrast. Dashed outlines indicate significant clusters where the empirical generalization is smaller than the expected generalization (*P* < 0.05, cluster permutation based on paired t-statistics, one-tailed, *N* = 19). **(H)** Relative cross-temporal generalization, as in panel D.

For spatially close representations, we found significant pattern generalization in a large fraction of simulations. While this percentage decreased for more distant representations, it was still higher than the expected false positive rate even at large distances (Fig. 4B). The severity of this effect increased with higher signal-to-noise ratios (SNR). As SNR was defined to correspond to the average cross-validated Mahalanobis distance, the results of this simulation can provide a guideline for the expected percentage of false positives in empirical data. For example, two neural representations 50 mm apart with realistic neural information corresponding to Mahalanobis distances of 1 each (see Fig. 5) would be expected to show significant spurious pattern generalization in 70% of experiments with 1000 trials and 19 participants.

We next used the measure of expected pattern generalization under the assumption of identity to assess the strength of spurious pattern generalization between orthogonal representations, in the extreme case of perfect stability and uniformity. To do so, we computed the expected pattern generalization values between representations in each pair of areas. We then computed the relative strength of spurious pattern generalization compared to this expectation under the assumption of identity. Spurious pattern generalization was markedly lower than expected if representations were identical (Fig. 4C). Only for representations within one area and in high SNR conditions, spurious pattern generalization reached relative values of about 0.7, whereas with increasing distance and very low SNR, relative pattern generalization values quickly dropped.

It thus appears to be highly unlikely for pattern generalization values to be spurious, while at the same time not being significantly lower than the expected pattern generalization. To quantify this, we determined the proportion of simulations in which pattern generalization between orthogonal representations was significantly different from 0, but not significantly different from its expected value under identity (Fig. 4D). Indeed, when applying this additional criterion, the fraction of false positives markedly decreased. For higher SNRs, as well as for any but the smallest spatial distances between representations, this reduced the number of false positives to the expected rate or below. This suggests that at least high pattern generalization corresponding to identical or close to identical patterns can serve as a reliable indicator of overlapping or spatially very close representations at the underlying neuronal level.

### 2.5 Example application: contrast and coherence information in MEG

To illustrate the considerate application of pattern generalization analyses to neural activity, we analyzed an MEG dataset recorded during the performance of a motion-direction discrimination task (Pellegrini et al., 2020) (Fig. 5A and B). Briefly, participants viewed dynamic random dot patterns presented either in the left or the right visual hemifield. Stimuli were either upwards- or downwards-moving and differed in their luminance contrast. We used the cross-validated Mahalanobis distance to compute neural information about stimulus contrast. Importantly, we also applied several pattern generalization analyses. First, we computed the pattern generalization between the contrast of stimuli presented in the left and right visual hemifields. Secondly, we applied a cross-temporal generalization analysis, using all pairs of time points within the trial to compute the generalization of contrast representations. In all cases, we used standard t-statistics and cluster permutation tests to assess statistical significance.

Upon stimulus presentation, there was significant contrast information (Fig. 5C, blue and violet traces, *P* < 0.05, one-tailed), both when stimuli were presented in the left and in the right hemifield. We found significant positive pattern generalization between the contrast of stimuli presented in the left and right hemifields (Fig. 5C, red trace, *P* < 0.05, two-tailed). Furthermore, there was a significant cross-temporal generalization of contrast information between a wide range of time points (Fig. 5E, *P* < 0.05, two-tailed).

Did these significant pattern generalization results reflect a true overlap of the underlying neural populations representing contrast in both hemifields and at different time points? To aid our interpretation, we first computed the expected pattern generalization under the assumption of identical representations (Fig. 5D, green trace).

Pattern generalization of contrast between stimuli shown in the left and right hemifields was significantly smaller than expected for identical representations throughout the trial (Fig. 5C, green bar above traces, *P* < 0.05, paired, one-tailed). Notably, pattern generalization and expected pattern generalization followed different time-courses. This can most easily be seen in the ratio between both values (Fig. 5D). While pattern generalization was initially low, it reached a relatively high level close to the expected value later in the trial. This suggests that, while early contrast information was likely driven by spatially selective circuits, it was later supported by populations that at least partially generalized across hemifields. Crucially, the significant but low-magnitude pattern generalization early in the trial should not be taken as conclusive evidence of overlapping populations representing contrast in both hemifields. Finally, the cross-time analysis indicated that contrast representations were significantly dynamic after stimulus onset, but largely stable during the sustained presentation of the stimulus (Fig. 5E–H).

Taken together, these empirical results provide both examples of pattern generalization that may well be spurious (initial response), and of pattern generalization values that are sufficiently high to warrant the careful conclusion of overlapping underlying representations (late sustained response).

## 3 Discussion

Multivariate decoding and encoding methods have become standard tools in neuroscience. As is true for any method, the promise they afford comes at the cost of hidden assumptions and interpretational pitfalls when not applied carefully. Several such issues have been described, and need to be taken into account when using or interpreting these analyses (Carlson et al., 2018; Driel et al., 2021; Hebart and Baker, 2018; Quax et al., 2019). Here, we shed light on the properties of pattern generalization, especially when applied to neural mass signals. Importantly, our results question the naïve interpretation of significant pattern generalization results as direct evidence of overlapping neural representations.

### 3.1 Spurious pattern generalization in multiple measurement modalities

While we focused on the case of large-scale non-invasive electrophysiological data gained from EEG or MEG, the issues that we presented apply beyond these methods. Although the specific limits of interpretability are determined by signal properties such as the spatial scale, any neural mass data involving at least some degree of signal mixing will face similar issues. Most notably, this includes fMRI data, as well as invasive population electrophysiology such as using local field potentials or electrocorticography.

These problems of pattern generalization interpretability are closely related to the spatial resolution of a measurement method. However, while for many other applications, the most relevant index of spatial resolution is localization accuracy, what matters in the case of pattern generalization is the cross-talk between neural sources. In the case of EEG or MEG, the cross-talk function between two sources may not even reach zero at the largest spatial distances, thus resulting in the possibility of spurious pattern generalization between representations in far-apart brain areas when SNR is sufficiently high.

### 3.2 The effect of high-pass filtering on temporal generalization

It has been noted that the use of high-pass filters on time series data can lead to spurious decoding (Driel et al., 2021). This can cause an additional complication: due to the sign reversals in the impulse response functions of commonly applied filters (e.g. Butterworth), spurious temporal generalization may seemingly reveal pattern reversals. This effect may be largely responsible for the widespread phenomenon of below-chance cross-time generalization (King and Dehaene, 2014; Vidaurre et al., 2021; Weisz et al., 2020). The interpretation of such below-chance temporal generalization results should thus be handled with extreme care, and always complemented by a discussion of potential filtering confounds.

### 3.3 Choosing a pattern generalization algorithm

For most of this manuscript, we have used the term *pattern generalization* to group together a large number of methods based on both linear classification algorithms as well as on cross-validated distance measures such as the cross-validated Mahalanobis distance (Diedrichsen et al., 2016; Walther et al., 2016) or cross-validated MANOVA (Allefeld and Haynes, 2014; Christophel et al., 2018). While these methods differ in some of their properties, they all share the fundamental issue of spuriously similar representations due to signal mixing.

Nonetheless, potential mitigation strategies and consequently the interpretability of pattern generalization results depend on the specific method used. First, classification accuracy suffers from nonlinearities and ceiling effects. The relationship to an underlying effect size is therefore not straightforward. This, secondly, also results in potential asymmetries of cross-classification. While such asymmetries have been interpreted as pointing towards meaningful physiological phenomena, they simply reflect differences in signal to noise ratio (van den Hurk and Op de Beeck, 2019). When assessing the similarity of representations between contexts, this constitutes a confound making it more difficult to interpret the magnitude of cross-classification values. Taken together, these points hinder the estimation of the expected cross-classification between identical representations.

Here, we therefore chose to use the cross-validated Mahalanobis distance; a distance measure that accurately reflects the separation between classes in an unbounded way, which enables the interpretability of pattern generalization values. The same also holds for other distance measures that are symmetric and unbiased, such as Euclidean distances or the cross-validated MANOVA (Allefeld and Haynes, 2014).

### 3.4 Can expected pattern generalization be estimated on population averages?

Throughout this article, we computed the expected pattern generalization under the assumption of identical representations on the level of single experimental repetitions or participants. This is because the expected pattern generalization should be estimated at the same level at which the multivariate pattern analysis itself is performed: if, for example, decoding is performed in individual subjects before then submitting the single subject results to a population level statistical test, the same procedure should usually be followed for the expected pattern generalization. The reason for this is that the non-linear computation of the expected pattern generalization and the linear averaging of single subject decoding values do not commute. When computing the expected pattern generalization between two representations based on population-averaged neural information, any across-subject variability in the ratio between the neural information about both representations would thus result in an over-estimation. Consequentially, any statistical test against the null hypothesis of identical representations would be prone to false positives.

### 3.5 Removing uniform responses

Here, we describe the uniformity of multivariate pattern differences as a factor contributing to spurious pattern generalization. This uniformity is closely related to similar concepts such as univariate responses, or overall activation differences between classes. The interpretation and handling of such uniform responses has been a matter of debate in the context of decoding analyses, and it can be helpful to distinguish decodability arising from response differences shared across a population from those arising from more fine-grained patterns (Hebart and Baker, 2018).

A strategy to both identify and exclude effects based on overall responses is the subtraction of an estimate of the shared pattern before the application of multivariate pattern analysis. In principle, the subtraction of shared response patterns could suppress uniformity as defined here. If successful, this would potentially mitigate the spurious pattern generalization caused by uniformity. This would however also entail a loss of sensitivity: due to population mixing, even fine-grained neuronal response patterns can appear uniform across sensors, and would thus be removed.

More importantly, methods to remove shared response patterns rest on strong assumptions, such as the absence of any additional, class-independent neural responses (Hebart and Baker, 2018). As these assumptions are difficult to verify, and are likely rarely met, the removal of shared response patterns may not only suppress, but also introduce spurious pattern generalization. Thus, while it may be interesting to assess the effect of shared pattern removal in a given dataset, it cannot be seen as a foolproof tool to mitigate spurious pattern generalization caused by uniformity. Nonetheless, future work in this direction may be fruitful to increase the interpretability of pattern generalization results.

### 3.6 Recommendations

Assessing the similarity of neural representations using pattern generalization remains an important analysis method. Even when using population measurement techniques that are not optimal for drawing conclusions at the neural level, pattern generalization analyses can facilitate meaningful scientific insights when the described problems are taken into account. To aid researchers in the application of pattern generalization analyses, we provide a list of recommendations. When followed, these should enable reliable inferences about the generalization of neural representations from neural mass data:

(1) Merely significant pattern generalization of population signals should not be interpreted as strong evidence of overlapping neural representations.
(2) Pattern generalization analyses should always be accompanied by an analysis of the neural information within each context to provide a reference for the strength of generalization that can be expected. If an unbiased distance measure is used, such as the cross-validated Mahalanobis distance, this expectation can be formalized as the expected pattern generalization under the assumption of identical representations. The relative strength of pattern generalization, compared to this expectation, constrains possible explanations: values close to the expectation indicate that both representations are indistinguishable given the properties of the data, and therefore either overlapping or spatially so close that their activation elicits identical measurement patterns.
(3) Pattern generalization can be tested against the null hypothesis of perfect stability. Interpretations are valid even for the underlying neural level. Thus, neural mass data can reliably be used to infer that representations are not identical, or dynamic in time.
(4) Reasonable assumptions about the spatial scale, stability and directional bias of the underlying representations can increase the interpretability of pattern generalization results. If there are good reasons to assume that circuit level representations are independent across replicates, even small pattern generalization values provide evidence for a representational overlap.
(5) Even in situations when pattern generalization itself cannot be meaningfully interpreted, it may be possible to make inferences based on condition differences in pattern generalization. This strategy would be valid when none of the confounding factors underlying spurious pattern generalization is expected to vary across conditions, such that true differences in generalization likely underlie the observed effects.

In sum, we show that, contrary to common practice, the mere presence of statistically significant pattern generalization in data measured on the population level does not allow strong inferences about the orthogonality of the underlying circuit-level representations. We argue that, with appropriate precautions, the pattern generalization framework can nonetheless be used to gain valuable insights into the neural mechanisms shared between contexts.

## Data and code availability statement

Data and code will be made available by the authors upon request.

## CRediT authorship contribution statement

**Florian Sandhaeger:** Conceptualization, Formal analysis, Writing – original draft, Writing – review & editing. **Markus Siegel:** Conceptualization, Writing – review & editing, Supervision, Resources, Funding acquisition.

## Declaration of Competing Interest

The authors declare no competing interests.

## Data Availability

Data will be made available on request.

## Appendix A

### A measure of expected pattern generalization

Let d1 be the true, noise-normalized pattern difference between two classes A and B, and d2 be the pattern difference in a different context. Then the true Mahalanobis distances between the classes are:

$$M1=d1*d1^{T}$$

$$M2=d2*d2^{T}$$

The pattern generalization between contexts 1 and 2 is then given by

$$M12=d1*d2^{T}$$

Depending on the angle between d1 and d2, this true pattern generalization may be either positive or negative, indicating a pattern reversal. Under the null hypothesis of perfectly identical patterns in the two contexts, the pattern differences between classes A and B are invariant up to a scaling factor:

d2=c*d1

Thus, the expected pattern generalization under stability can be calculated as

$$\begin{array}{ccc} M12_{S} & = & d1*{\left(c*d1\right)}^{T}=c*M1=\sqrt{c^{2}*M1^{2}}=\sqrt{M1*c^{2}*d1*d1^{T}}\\ & = & \;\sqrt{M1*d2*d2^{T}}=\;\sqrt{M1*M2} \end{array}$$

When using real data, we do not have access to the true pattern differences and have to use cross-validation to avoid finding spurious information and determine the empirical cross-validated Mahalanobis distances M1_E_, M2_E_ and M12_E_. However, the cross-validated Mahalanobis distance provides an unbiased estimator, such that E[M1_E_] = M1, E[M2_E_] = M2, and E[M12_E_] = M12. Empirical Mahalanobis distances can, when there is little signal, become negative. If this happens for either M1 or M2, the expected pattern generalization would become complex-valued. Avoiding this by limiting the values of M1 and M2 to the positive range would introduce a positive bias: even in the case of low SNR, when M1 and M2 are expected to scatter around 0, the expected pattern generalization would always be positive. Thus, even when both representations are identical, empirical pattern generalization would often be significantly smaller than the expected pattern generalization. To avoid this positive bias, we multiply with the product of the signs of M1 and M2. Thus, we define the empirical expected pattern generalization under the assumption of identity as

$$M12_{SE}=\;\sqrt{\left|M1_{E}*M2_{E}\right|}*sign\left(M1_{E}\right)*sign\left(M2_{E}\right)$$

This measure provides a lower bound for the true expected pattern generalization: For an infinite signal to noise ratio, M1_E_ and M2_E_ are always positive. We can thus simplify the expression and use the Cauchy-Schwarz inequality to show that

$$\begin{array}{ccc} E[M12_{S\infty ]} & = & \;E\left[\sqrt{M1_{E}*M2_{E}}\right]\leq \;\sqrt{E\left[M1_{E}\right]*E\left[M2_{E}\right]}\\ & = & \;\sqrt{M1*M2}=M12_{S} \end{array}$$

With lower signal to noise ratio, and partially negative distributions of M1_E_ and M2_E_, the square root in the first part of the expression becomes positively biased. This is counteracted by the second part. In the extreme, with M1_E_ or M2_E_ symmetrically distributed around zero, M12_SE_ will also be symmetrically distributed around zero.

As M12_SE_ is a lower bound of the true expected pattern generalization under identity, we can use it to test against the null hypothesis of identical representations: if the empirical pattern generalization M12 is smaller than M12_SE_, the pattern differences in the two contexts are not identical.

Notably, the expected pattern generalization under identity defined here is closely related to an SNR-corrected correlation between the pattern differences d1 and d2 (Siems et al., 2016).

### MEG analysis and source simulation

We used data from a previously published study (Pellegrini et al., 2020) to provide an example cross-decoding analysis. 19 Participants performed a motion discrimination task while MEG was recorded using a 275-channel system (Omega 2000, CTF Systems Inc.). Participants provided written informed consent prior to the start of the experiment. The study was conducted in accordance with the Declaration of Helsinki and was approved by the ethical committee of the Medical Faculty and University Hospital of the University of Tübingen. Detailed procedures are reported elsewhere (Pellegrini et al., 2020). Briefly, on each trial, after a 500ms fixation baseline, participants viewed a dynamic random-dot pattern presented for 1000ms either in the left or right hemifield (10 º eccentricity, 12 º stimulus diameter). After a variable delay (300-600ms), a brief dimming of the fixation cross served as a go cue in response to which participants had to indicate whether they saw upward or downward motion using a button press.

All stimuli were either upward- or downward-moving and had varying motion coherence (12%, 56% and 100%) and luminance contrast (20%, 60% and 100%).

For the present analysis, we omitted the intermediate levels of both coherence and contrast, keeping only those trials were both features were either at the lowest or the highest level. We used the data of all 18 out of the 19 participants who either finished the total 900 trials, or at least as many as required to counterbalance all conditions in the pattern generalization analysis. MEG data was high-pass filtered at 0.01 Hz, low-pass filtered at 20 Hz and resampled to 50 Hz.

We then used cross-validated Mahalanobis distances to quantify the amount of information present in the data about stimulus contrast. After baseline-correcting each trial and estimating a common noise covariance matrix across all conditions for each time-point, we computed cross-validated Mahalanobis distances using 5-fold cross-validation after making sure trials of all combinations of contrast, coherence, hemifield and motion direction were present equally often. All analyses were performed separately for stimuli presented in the left and right hemifields, and subsequently averaged or contrasted.

In addition to these neural information analyses, we also employed pattern generalization analyses. We assessed generalization, first, across space, using the contrast of stimuli presented on one side on the training data, and the contrast of stimuli presented on the other side on the test data. Secondly, we assessed temporal generalization, using one time point as training data another time point as testing data.

For all pattern generalization analyses, we computed the measure of expected pattern generalization for identical representations described above. Using cluster-based permutation tests, we then assessed two hypotheses: first, whether pattern generalization was significantly different from 0, and secondly, whether pattern generalization was significantly smaller than expected for identical representations.

Each participant also took part in a structural MRI measurement. We used T1 images to construct individual forward models projecting from a set of 457 sources uniformly distributed over the cortical surface to 272 MEG sensors. These forward models were then used to project simulated source level data to the sensor level.

### Software

All analyses were performed in MATLAB, using custom code as well as the Fieldtrip (Oostenveld et al., 2011) and SPM toolboxes.
